# Lack of in vivo mutagenicity of carbendazim in the liver and glandular stomach of MutaMice

**DOI:** 10.1186/s41021-024-00299-4

**Published:** 2024-02-20

**Authors:** Takako Iso, Kenichiro Suzuki, Yasumasa Murata, Nozomu Hirose, Takaaki Umano, Katsuyoshi Horibata, Kei-ichi Sugiyama, Akihiko Hirose, Kenichi Masumura, Mariko Matsumoto

**Affiliations:** 1 Division of Risk Assessment, National Institute of Health Sciences, Kanagawa, Japan; 2 Genotoxicology Laboratory, BioSafety Research Center Inc., Shizuoka, Japan; 3 Division of Genetics and Mutagenesis, National Institute of Health Sciences, Kanagawa, Japan; 4 Chemicals Evaluation and Research Institute, Tokyo, Japan

**Keywords:** Carbendazim, In vivo mutagenicity, Transgenic rodent gene mutation assay

## Abstract

**Background:**

Carbendazim (methyl 2-benzimidazolecarbamate, CASRN: 10605-21-7) exhibits spindle poisoning effects and is widely used as a fungicide. With respect to genotoxicity, carbendazim is deemed to be non-mutagenic in vitro, but it causes indicative DNA damage in vivo and chromosome aberrations in vitro and in vivo. In this study, we examined the mutagenicity of carbendazim in vivo.

**Results:**

MutaMice were treated with carbendazim orally at doses of 0 (corn oil), 250, 500, and 1,000 mg/kg/day once a day for 28 days. A *lacZ* assay was used to determine the mutant frequency (MF) in the liver and glandular stomach of mice. MutaMice were administered up to the maximum dose recommended by the Organization for Economic Co-operation and Development Test Guidelines for Chemicals No. 488 (OECD TG488). The *lacZ* MFs in the liver and glandular stomach of carbendazim-treated animals were not significantly different from those in the negative control animals. In contrast, positive control animals exhibited a significant increase in MFs in both the liver and glandular stomach.

**Conclusions:**

Carbendazim is non-mutagenic in the liver and glandular stomach of MutaMice following oral treatment.

## Introduction

Carbendazim belongs to the benzimidazole fungicide family. Its mode of action involves binding to *β*-tubulin to disrupt microtubule assembly and inhibit cell division. The antimicrobial efficacy of carbendazim has industrial applications, where it is incorporated as a preservative into paints, films, plastic, concrete, and textiles. It is also sprayed foliar in a wide variety of crops as a pesticide [1, 2].

In Japan, carbendazim is used as a preservative during manufacturing and occupational workers are believed to be exposed to carbendazim. It is listed as an additive on a positive list of the food apparatus, containers, and packaging under “Food Sanitation Act” of Japan. Consumers may ingest small amounts of carbendazim that leaches from these products to food. In developing countries, benzimidazole agents have been indiscriminately used on farmland [3, 4]. A survey in Nepal concluded that on average, 1.91 kg active ingredient/ha of carbendazim is used in vegetable cultivation, although the recommended dose is 0.050–0.250 kg active ingredient/ha [3]. Concerns have been raised that long-term carbendazim exposure may cause DNA damage with insufficient risk regulations and little health protection [3, 5].

The safety of carbendazim was evaluated by the World Health Organization (WHO) in 1993 [6], the FAO/WHO Expert Committee on Pesticide Residues (JMPR) in 1995 and 2005 [2, 7], and European Food Safety Authority (EFSA) in 2010 and 2012 [8, 9]. In 2019, the EU released an assessment report [1]. Most of the data reviewed by the risk assessment bodies include unpublished studies and information could only be obtained from secondary sources.

The biological fate of carbendazim has been described in rodents and livestock. In rats, the xenobiotic profile of carbendazim following oral gavage revealed > 85% gastrointestinal absorption calculated from urinary excretion [10]. Most of the administered radioactivity was excreted within 72 h, primarily in the urine. The excretion half-life is approximately 12 h for both sexes, with residual radioactivity detected only in the liver and carcass [1, 11, 12]. In rats, the proposed biotransformation pathway of carbendazim involves oxidization of the phenyl ring, resulting in the formation of methyl (5-hydroxy-1 H-benzimidazol-2-yl)-carbamate (5-HBC) and 5,6-dihydroxy-carbendazim, which are subsequently conjugated with glucuronic acid and sulfate [10–13]. The same metabolites were found in mouse urine [11, 14]. Based on a residue analysis in livestock (lactating cow, nonlactating goat, and laying hen), radioactivity in the edible part was marginal after discontinuing the treatment [6, 15–18]. Carbendazim was extensively biotransformed through oxidation, to form the same metabolites produced in rats and mice. In addition, methyl (4-hydroxy-1 H-benzimidazol-2-yl)-carbamate was detected in livestock [15–18]. Thus, the biotransformation of carbendazim is not markedly different between species.

Under short-term oral doses of carbendazim, the liver and testis are toxicological targets [1]. Major findings include increased liver weight, decreased testis weight, and azoospermia. Subchronic toxicity studies in rats and dogs, mostly conducted between the 70s and 80s, showed similar effects [11]. Based on hepatotoxicity and testicular toxicity, the no-observed-adverse-effect level (NOAEL) was 163 and 2.7 mg/kg/day in rats and dogs, respectively [11, 19, 20].

In orally dosed long-term toxicity and carcinogenicity studies in mice, rats, and dogs, the liver is the target organ. Carbendazim induces centrilobular hypertrophy (rats and mice) and changes in hematology without histopathological findings (dogs) [1]. Based on hepatotoxicity, the derived NOAEL was 22 mg/kg/day (rat) and 2.6 mg/kg/day (dog) [21]. Neoplastic changes in the liver were observed in two sensitive mouse strains (CD-1 and Swiss), but not in NMRKf mice [11, 22–24]. The combined incidence of hepatocellular adenoma and carcinoma in CD-1 mice increased significantly, and the postulated NOAEL was < 81 mg/kg/day [11, 23, 24]. In Swiss mice, neoplastic liver nodules in females and hepatoblastoma in males have been reported, with a NOAEL of 22.5 mg/kg/day [11, 24, 25]. Risk assessment bodies suggest that liver tumors in susceptible mouse strains are not relevant to humans [1, 6, 8]; however, carbendazim caused hepatic aneuploidy in ddY mice [26]. Somatic tumor cells are often aneuploid with chromosome instability and the correlation between aneuploidy and tumorigenesis is still controversial [27–32]. Therefore, the risk of carcinogenesis from carbendazim exposure cannot be excluded.

Gene mutation studies in *Salmonella typhimurium* and *Escherichia coli* have reported conflicting results [11, 33–38]. Positive results were believed to be derived from the impurities 2, 3-diaminophenazine, 3-amino-2-hydroxyphenazine, and an unidentified chemical. Highly purified carbendazim (purity: > 99.50%) did not cause mutagenicity in the presence of S9 mix with *Salmonella typhimurium* strains TA98 and TA1538. In contrast, two different batches of technical carbendazim (purity: 97.8% and 90.11%) were positive at levels higher than 5,000 µg/plate [11, 39]. Based on these data, carbendazim was deemed to non-mutagenic in vitro [1, 6, 8]. Positive results in TA98 and TA1535 were reported for three other batches of carbendazim (purity: > 99%) in an unpublished study [11], and it did not cause gene mutations in mammalian cell cultures (HPRT test with Chinese hamster ovary cells, mouse lymphoma tests with L5178Y *TK*+/− cells) [11, 40–42]. In contrast, many studies have demonstrated that carbendazim causes chromosome aberrations in vitro and in vivo [43–50]. Numerical chromosome aberrations are well-defined by identifying kinetochore-positive micronuclei with immunofluorescent antibodies [47] and by examining chromosomes using fluorescence in situ hybridization (FISH) with centromere probes [45, 46]. In mice, carbendazim-induced micronuclei were detected in bone marrow after a single oral dose at 1,646 mg/kg [47], in hepatocytes after a single oral dosing 125–1,000 mg/kg [26], and in the gut following a single oral dose at 500 and 1,000 mg/kg [51]. In rats, micronuclei were detected in the bone marrow after one or two oral doses at 150 mg/kg [52] and in the spermatids after a single oral dose of 100 mg/kg [53]. Of note, the comet assay detected DNA damage that may be converted into a heritable mutation in the lymphocytes and hepatocytes of mice treated with 20 µM drinking water and 20 mg/kg oral carbendazim for subchronic treatment [5, 54].

Carbendazim is deemed to be non-mutagenic in vitro based on unpublished data from secondary sources [1, 6, 8]. Carbendazim primarily targets the liver and testis and induces hepatic tumors in some mouse strains; however, the correlation between aneuploidy and tumorigenicity has not been firmly established [55]. In contrast, long-term persistent genotoxic effects are of concern [4, 5]. In this study, we performed transgenic rodent gene mutation assays to determine whether carbendazim-mediated genotoxicity induces mutations in vivo. For the analysis, we selected the liver, which is a major organ to which carbendazim is distributed. It is for metabolism and associated with tumorigenesis and exhibits high sensitivity in this assay. We also evaluated the glandular stomach, which is directly exposed by a gavage route.

## Results

Male MutaMice were orally treated with carbendazim at doses of 0 (corn oil), 250, 500, and 1,000 mg/kg/day once a day for 28 days. Tissues were collected three days after the final treatment. No mortality, clinical signs of toxicity, or treatment-related changes in body weight were observed in either group. In addition, no gross pathological abnormalities were evident in the liver or glandular stomach at necropsy (data not shown).

A *lacZ* assay was performed to estimate MFs in the liver and glandular stomach (five mice per group). Tables 1 and 2 show the *lacZ* MFs from the negative control, carbendazim-treated, and positive control (*N*-ethyl-*N*-nitrosourea (ENU)-treated animals) tissues. In the liver, *lacZ* MFs (×10^− 6^) were 41.2 ± 12.8, 39.2 ± 9.0, and 45.7 ± 15.7 (mean ± SD), respectively, in the 250, 500, and 1,000 mg/kg/day treatment group, which were not significantly different from that of the negative control animals (40.9 ± 11.6). In contrast, *lacZ* MF (×10^− 6^) in the positive controls (108.1 ± 23.8) was significantly increased compared with that in the negative control animals (*p* < 0.05, Student’s *t*-test).

**Table 1:** Mutant frequencies in the liver of MutaMice treated with carbendazim

Substance	Dose (mg/kg/day)	Animal ID	No. of total plaques	No. of mutants	Mutant frequency (×10^− 6^)	Mean ± S.D. (×10^− 6^)
Corn oil	0	3001	417,600	11	26.3	
		3002	363,600	12	33.0	
		3003	566,100	30	53.0	40.9 ± 11.6
		3004	466,200	24	51.5	
		3005	562,500	23	40.9	
Carbendazim	250	3101	473,400	19	40.1	
		3102	828,000	44	53.1	
		3103	730,800	16	21.9	41.2 ± 12.8
		3104	399,600	21	52.6	
		3105	626,400	24	38.3	
	500	3201	432,000	17	39.4	
		3202	343,800	11	32.0	
		3203	438,300	20	45.6	39.2 ± 9.0
		3204	437,400	22	50.3	
		3205	417,600	12	28.7	
	1,000	3301	636,300	37	58.1	
		3302	558,900	17	30.4	
		3303	495,000	16	32.3	45.7 ± 15.7
		3304	304,200	20	65.7	
		3305	478,800	20	41.8	
ENU	100	3401	366,300	37	101.0	
		3402	505,800	45	89.0	
		3403	430,200	62	144.1	108.1 ± 23.8*(S)
		3404	549,900	48	87.3	
		3405	302,400	36	119.0	

Corn oil: Negative control (10 mL/kg)

ENU: Positive control (*N* -ethyl-*N* -nitrosourea, 10 mL/kg, i.p., dose once daily for 2 days, expression period; 10 days)

*: Significant difference from negative control (*p* < 0.05)

(S): Student’s *t* test

**Table 2:** Mutant frequencies in the glandular stomach of MutaMice treated with carbendazim

Substance	Dose (mg/kg/day)	Animal ID	No. of total plaques	No. of mutants	Mutant frequency (×10^− 6^)	Mean ± S.D. (×10^− 6^)
Corn oil	0	3001	608,400	16	26.3	
		3002	561,600	21	37.4	
		3003	354,600	19	53.6	40.6 ± 10.4
		3004	360,900	14	38.8	
		3005	318,600	15	47.1	
Carbendazim	250	3101	330,300	10	30.3	
		3102	722,700	35	48.4	
		3103	539,100	23	42.7	38.9 ± 10.5
		3104	474,300	12	25.3	
		3105	333,900	16	47.9	
	500	3201	360,900	16	44.3	
		3202	311,400	18	57.8	
		3203	593,100	26	43.8	41.1 ± 11.9
		3204	827,100	23	27.8	
		3205	538,200	17	31.6	
	1,000	3301	344,700	11	31.9	
		3302	727,200	33	45.4	
		3303	330,300	12	36.3	37.7 ± 7.2
		3304	399,600	18	45.0	
		3305	398,700	12	30.1	
ENU	100	3401	358,200	129	360.1	
		3402	661,500	241	364.3	
		3403	311,400	122	391.8	396.1 ± 53.8*(AW)
		3404	582,300	218	374.4	
		3405	432,900	212	489.7	

Corn oil: Negative control (10 mL/kg)

ENU: Positive control (*N* -ethyl-*N* -nitrosourea, 10 mL/kg, i.p., dose once daily for 2 days, expression period; 10 days)

*: Significant difference from negative control (*p* < 0.05)

(AW): Aspin-Welch’s *t* test

In the glandular stomach, *lacZ* MFs (×10^− 6^) were 38.9 ± 10.5, 41.1 ± 11.9, and 37.7 ± 7.2, respectively, in the 250, 500, and 1,000 mg/kg/day treatment group, which was not significantly different from that of the negative control animals (40.6 ± 10.4). In contrast, *lacZ* MF (×10^− 6^) in the positive controls (396.1 ± 53.8) was significantly increased from that in the negative control animals (*p* < 0.05, Aspin–Welch’s *t*-test).

## Discussion

In this study, we determined whether in vivo genotoxicity, such as DNA damage, micronucleus formation, and aneuploidy caused by carbendazim, result in gene mutations *in vivo.* We set the doses at 250, 500, and 1,000 mg/kg/day based on the results of range-finding toxicity studies, in which 1,000 mg/kg/day was the maximum dosage recommended by the guidelines. No toxicological changes in body weight, gross observations, or necropsy findings in the liver and glandular stomach were observed in the MutaMice. The xenobiotic profiles of mice and rats are comparable and gastrointestinal absorption after oral dosing is rapid and high [10, 11, 14]; therefore, carbendazim was assumed to be systemically absorbed by the gastrointestinal tract and distributed via the liver [10–12]. Genotoxicity studies in mice revealed colon and bone marrow micronuclei after a single oral dose of 500 and 1,646 mg/kg, respectively [47, 51]. DNA strand breaks were observed in male Swiss mouse lymphocytes after a 90-day treatment of 5.4 mmol/kg/day carbendazim in drinking water (approximately 1 mg/kg/day) [5]. The mice exhibited DNA damage, but no change in body weight, food and water consumption, or gross behavior [5]. Based on our results and other studies, we hypothesize that carbendazim reaches the glandular stomach and liver without showing visible signs of toxicity.

Conflicting results can occur in bacterial assays because of mutagenic contaminants; however, carbendazim itself did not show mutagenic potential according to several risk assessment reports [1, 2, 6, 8]. Although a risk assessment decision was publicly announced, the underlying data were not published. Therefore, we could not evaluate these studies. Here, we show that the MFs in the liver and glandular stomach tissue of carbendazim-treated animals were similar to those of the negative control animals. This confirms that carbendazim did not cause mutations in the liver and glandular stomach in vivo, and the development of liver tumors may occur through a non-mutagenic mode of action. Tumorigenic responses to carbendazim were observed in CD-1 and Swiss mice. In long-term toxicity and carcinogenicity studies, CD-1 and Swiss mice treated with carbendazim showed hepatic alterations, such as increased weight, centrilobular hepatocellular swelling or necrosis, focus/foci of eosinophilic cellular alterations, and nodular hyperplasia [11, 23, 24]. Benomyl, a benzimidazole family fungicide and carbendazim precursor, caused hepatic tumors in CD-1 and Swiss mice as well as hepatocellular toxicity followed by cell proliferation and cytochrome P450 induction in a subchronic (28-day) feeding study [56, 57]. Thus, benomyl and carbendazim induce hepatic neoplasms after hepatic alterations [56]. Oral exposure to carbendazim resulted in hepatic numerical aberrations in ddY mice bearing resected livers [26]. Carbendazim-induced chromosomal instability and hepatotoxicity may be one possible mechanism of tumorigenesis. The role of aneuploidy in carcinogenesis has not been fully established and it may not have a primary causative role [32]. Therefore, further mechanistic studies on carbendazim-mediated carcinogenesis are warranted.

Aneuploidy in germ cells results in infertility and pregnancy loss [58, 59]. Carbendazim treatment did not cause any histopathological changes in female reproductive organs and had no estrogenic or anti-estrogenic effect on uterine weight [4]. However, carbendazim reaches the oocytes and induces aneuploidy, suggesting a correlation between aneugenic oocytes and implantation loss [48, 50, 60, 61]. Numerical chromosome aberrations in the spermatids of carbendazim-treated rats suggest concomitant testicular toxicity [62, 63]. With respect to reproduction and developmental toxicity, carbendazim caused testicular toxicity, characterized by vacuolization of Sertoli cells as well as sloughing and elongation of spermatids and spermatocytes [63–67]. In addition, carbendazim altered serum LH, FSH, testosterone, and GnRH levels [68, 69]. This suggests that carbendazim-mediated reproductive and developmental toxicity may also have an endocrine mode of action, although contradictory results have been reported [4]. Our study was designed to examine the mutagenic potency of carbendazim in somatic cells. Meanwhile, OECD TG488 requires prolonged fixation time, such as 28 days of exposure and 28 days of fixation in germ cells [70, 71]. Further studies are needed to elucidate the overall mechanism of toxicity by carbendazim.

Our results provide convincing evidence that carbendazim is non-mutagenic and that a threshold mechanism may occur, which results in its toxicity and carcinogenicity. Because the dietary risk of carbendazim has not been evaluated in Japan, these results are meaningful for risk assessment and regulation.

## Conclusions

We performed transgenic rodent gene mutation assays using MutaMice to evaluate the mutagenicity of orally administered carbendazim. Although MutaMice were administered up to the maximum dose recommended by the OECD guidelines, *lacZ* MFs in the liver and glandular stomach of carbendazim-treated animals were not affected. Our results indicate that carbendazim is non-mutagenic in the liver and glandular stomach of MutaMice following oral exposure.

## Methods

This study was conducted by OECD TG 488 [72] at the BioSafety Research Center (BSRC; Shizuoka, Japan) in compliance with “Act on Welfare and Management of Animals” [73], “Standards relating to the Care and Keeping and Reducing Pain of Laboratory Animals” [74], “BSRC Guidelines for Animal Experimentation,” and “Act on the Conservation and Sustainable Use of Biological Diversity through Regulations on the Use of Living Modified Organisms” [75]. The study was approved by the BSRC Safety Management Regulations for Recombinant DNA Experiment.

### Test chemicals and reagents

Carbendazim (CASRN: 10605-21-7, Lot no. MKCM2970, purity: 97%) was purchased from Sigma-Aldrich Japan (Tokyo, Japan). ENU (CASRN: 759-73-9), a positive control, was purchased from Toronto Research Chemicals Inc. (Ontario, Canada). Corn oil was purchased from FUJIFILM Wako Pure Chemical Corp. (Osaka, Japan). Carbendazim was suspended in corn oil. The dosing formulations were stored at room temperature until use and used within 8 days after preparation. Stability and uniformity during storage were verified. ENU was dissolved in phosphate buffer (pH 6.0) before administration.

### Animals and breeding conditions

We purchased male and female CD2F1 mice as well as male MutaMice (CD2-LacZ80/HazfBR), aged 8 weeks, from Japan SLC, Inc. (Shizuoka, Japan) and Trans Genic Inc. (Fukuoka, Japan). Food (CRF-1, Oriental Yeast, Japan) and water were provided *ad libitum*. The animals were maintained at 20–26 °C, 35–70% relative humidity, 12-h light/dark cycle, and 12 air changes per hour. The animals were acclimatized for 8 days before beginning treatment.

### Dose selection

In a range-finding toxicity study, four groups of three male and three female CD2F1 mice were administered 30, 100, 300, or 1,000 mg/kg/day of carbendazim in a volume of 10 mL/kg once daily for 14 days. The highest dose was established based on OECD TG 488, and the four levels were divided by a geometric ratio of three. Body weight was recorded on days 1, 8, and 15 (next day of the last dosing) and mortality and clinical signs were checked daily. The surviving animals were euthanized with CO_2_ on day 15. There were no obvious sex differences in the range-finding toxicity study; therefore, only male mice were included in the main study. Mortality, clinical signs of toxicity, or treatment-related body weight changes were not observed up to the highest tested dose. We set the main study dosage at 0, 250, 500, and 1,000 mg/kg/day, up to the maximum dosage recommended by the guidelines for 28-day repeated administration.

### Treatments and tissue isolation

Four groups containing six male MutaMice each were administered 0 (vehicle: corn oil), 250, 500, and 1,000 mg/kg/day carbendazim at a constant volume of 10 mL/kg once daily for 28 days. The mice were euthanized three days after the final treatment. Body weight was recorded on days 1, 8, 15, 22, 29, and 31 (before tissue isolation), and mortality and clinical signs were checked daily. Positive controls were administered 100 mg/kg/day ENU by intraperitoneal injection on days 2 and 3 once daily. The mice were euthanized 10 days after the final treatment. Body weight was recorded on days 1 and 13 (before the tissue isolation). Mortality and clinical signs were checked daily in the positive control group.

The liver and stomach were isolated and a gross pathological examination was conducted. Two points of the left lateral lobe of the liver were hollowed out and frozen in liquid N_2_. The forestomach and glandular stomach were separated and the glandular stomach was frozen in liquid N_2_. Frozen samples were stored in an ultradeep freezer at −80 °C until further analysis.

### Genomic DNA isolation

Genomic DNA was isolated from the tissues of five animals in each group in ascending order of animal ID as described previously [76]. Briefly, frozen tissue was homogenized using a pestle in Dounce buffer and the homogenized tissue was transferred to an ice-cold centrifuge tube containing 0.5 mol/L sucrose. After centrifugation at 1,750 × *g* for 10 min, the supernatant was removed. Precipitated nuclei/cells were resuspended in 3 mL of Dounce buffer containing 0.002% RNase (NIPPON GENE Co., Ltd.), mixed with 3 mL of 0.2% proteinase K solution (FUJIFILM Wako Pure Chemical Co., Ltd.), and incubated at 50 °C for 2 h. The suspension was mixed with an equal volume of phenol/chloroform (1:1), rotated for 10 min, and centrifuged at 1,220 × *g* for 10 min. The aqueous layer was collected and extracted twice with phenol: chloroform. The aqueous layer was mixed with an equal amount of chloroform: isoamyl alcohol (24:1) and extracted in the same manner. Genomic DNA was precipitated by adding ethanol to the aqueous layer. The precipitated DNA was rinsed with 70% ethanol for 10 min, placed at room temperature, air-dried overnight, and dissolved in TE buffer (NIPPON GENE Co., Ltd.). The purified DNA was stored in a refrigerator NanoDrop (AGC TECHNO GLASS Co., Ltd.) was used to determine the DNA concentration.

### In vitro packaging

Lambda in vitro packaging reaction was performed for transgene rescue based on the Transpack instruction manual (Agilent Technologies, Transpack Packaging Extract Catalog #200,220, #200,221, and #200,223). Approximately 10 µL of genomic DNA (200–600 µg/mL) was gently mixed with the Transpack packaging reagent, incubated twice at 30 °C for 1.5 h, and mixed with 700 µL of SM buffer to stop the reaction.

### MF determination

A *lacZ* mutation assay was performed as previously described [76]. Briefly, a mixture of *E. coli* C (*lacZ*^*−*^, *gal E*^*−*^) cell suspension and the total packaged sample were incubated for 30 min. The rescued phages were absorbed into *E. coli.* An aliquot of this suspension was diluted and mixed with LB top agar for titer plates. The remaining cell suspension was mixed with LB top agar containing phenyl-β-D-galactoside for selection. Both plates were incubated overnight at 37 °C. MFs were calculated by dividing the total number of mutant plaques on the selection plates by the total number of rescued phages.

### Statistical analysis

Bartlett’s test was used to compare the homogeneity of variances across the groups. When homogeneity was detected, Dunnett’s test was used for analysis. Steel’s test was used for non-homogeneous data. Based on the results of an F-test, a Student’s *t*-test or Aspin–Welch’s *t*-test was used to compare MFs between negative and positive controls. The criterion for significance was set at 5% probability.

## Data Availability

All available data are presented in this article.

## Abbreviations

MF: mutant frequency
OECD: Organization for Economic Co-operation and Development
WHO: World Health Organization
JMPR: FAO/WHO Expert Committee on Pesticide Residues
EFSA: European Food Safety Authority
NOAEL: no-observed-adverse-effect level
ENU: *N*-ethyl-*N*-nitrosourea
LH: luteinizing hormone
FSH: follicle stimulating hormone
GnRH: gonadotropin-releasing hormone
